# Radiomics analysis of brain–tumor interface on multiparametric MRI for prediction of brain metastasis primary site

**DOI:** 10.1186/s13244-026-02464-5

**Published:** 2026-11-06

**Authors:** Mingchen Jiang, Bingzhu Han, Yufei Huang, Xinmeng Rong, Wenjie Zhao, Xueyan Ge, Ronghui Ju, Wenyan Jiang

**Affiliations:** 1 School of Intelligent Medicine, China Medical University, Shenyang, P.R. China; 2 Department of Radiology, The People’s Hospital of Liaoning Province, Shenyang, P.R. China; 3 School of Biomedical Engineering, Faculty of Medicine, Dalian University of Technology, Dalian, China; 4 Department of Scientific Research and Academic, Cancer Hospital of Dalian University of Technology, Liaoning Cancer Hospital & Institute, Cancer Hospital of China Medical University, Shenyang, China

**Keywords:** Brain neoplasms, Neoplasm metastasis, Magnetic resonance imaging, Radiomics, Tumor microenvironment

## Abstract

**Objectives:**

Brain metastasis (BM) frequently occurs in advanced cancer patients, and accurate identification of the primary origin is essential for personalized treatment. Conventional imaging often lacks sensitivity and specificity for BM source prediction. This study aimed to develop and validate a multiparametric MRI-based radiomics model emphasizing brain–tumor interface (BTI) features to improve noninvasive BM origin identification.

**Materials and methods:**

The primary cohort included 429 patients (740 BMs) from two centers, non-small cell lung cancer (NSCLC), small cell lung cancer (SCLC), breast cancer (BC), and gastrointestinal cancer (GIC), and an independent validation cohort of 33 patients (84 BMs) from a third center. All underwent pre-treatment 3.0 T MRI with T1-weighted contrast-enhanced (T1CE) and T2-weighted (T2W) sequences. Radiomic features were extracted from tumor core and BTI regions (2, 4, 6, 8 mm). Least absolute shrinkage and selection operator (LASSO) regression was used for feature selection, and radiomics signatures (RS) were evaluated using receiver operating characteristic (ROC) analysis.

**Results:**

The constructed RS-BTI4 based on the 4-mm BTI region generated the best overall performance, with area under the ROC curve (AUCs) in the training cohort were 0.855 (LC/NLC), 0.910 (SCLC/NSCLC), and 0.890 (BC/GIC); in validation cohort, 0.726, 0.856, and 0.809; and in external validation cohort, 0.739, 0.750, and 0.792, respectively. RS-BTI4 consistently outperformed models based on other BTI radial widths and the whole tumor region in identifying the primary origin of BM.

**Conclusion:**

BTI-derived radiomic features demonstrate strong potential for noninvasive identification of BM origin, with the 4-mm BTI region showing optimal predictive performance.

**Key Points:**

***Question*** Conventional MRI cannot reliably identify the primary origin of brain metastases, particularly when the primary tumor remains unknown at diagnosis.

***Findings*** The 4-mm brain–tumor interface model showed the most favorable overall performance, measured by area under the curve, across three binary classification tasks and validation cohorts.

***Critical relevance statement*** Integrating brain–tumor interface radiomics with multiparametric MRI may improve noninvasive estimation of BM origin and support more targeted diagnostic evaluation in clinical radiology.

**Graphical Abstract:**

**Figure:**
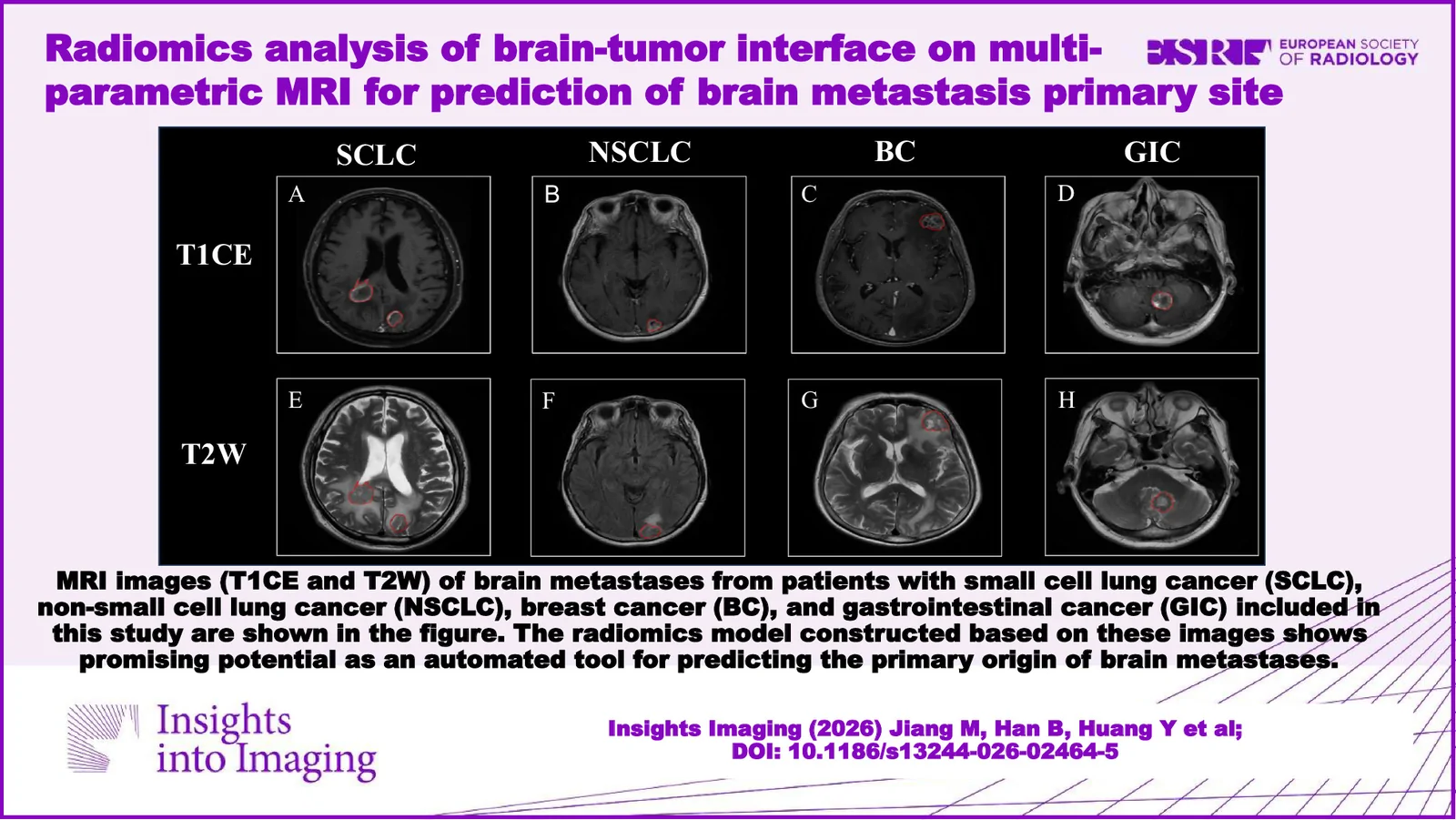

## Introduction

Brain metastasis (BM) is one of the most common neurological complications in patients with advanced cancer, with approximately 20% of cancer patients developing BM during disease progression [1, 2]. The most frequent primary sites of BM include non-small cell lung cancer (NSCLC) (35%–56%), small cell lung cancer (SCLC) (9%–20%), breast cancer (BC) (13%–30%), melanoma (MM) (6%–11%), and gastrointestinal cancers (GIC) (6%–9%), which together account for an estimated 67%–80% of all cases [3–5].

Although imaging modalities such as computed tomography (CT) and magnetic resonance imaging (MRI) are routinely used and are essential tools in the diagnosis and monitoring of BM, the substantial heterogeneity of BMs and their complex biological behavior significantly hinder the accurate identification of the primary tumor site based on visual analysis of imaging features [6]. In clinical practice, definitive determination of BM origin often requires procedures including positron emission tomography (PET) and histopathological biopsy. These approaches not only increase diagnostic complexity but are also associated with considerable costs and inherent procedural risks [7, 8]. Notably, even with comprehensive diagnostic assessments, the primary tumor site remains unidentified at the time of BM diagnosis in approximately 2%–14% of cases [9], and these patients usually have an unfavorable prognosis, characterized by markedly reduced survival and limited to palliative treatment options [10–12]. Accordingly, there is an urgent need to develop accurate and noninvasive imaging-based methods for predicting the primary origin of BM, in order to facilitate clinical decision-making and advance personalized therapeutic strategies [13].

In recent years, radiomics has emerged as a novel technique that enables comprehensive quantitative characterization of tumor morphology and microenvironmental heterogeneity from MRI images, thus providing new insights into tumor diagnosis, prognosis, and treatment planning [14, 15]. In the field of BM analysis, radiomics has facilitated the identification of diverse morphological patterns by quantitatively analyzing brain imaging data [16]. Recent studies have demonstrated that radiomics features derived from brain MRI can improve the prediction of BM origin [7, 17–19]. More recently, machine learning–based MRI radiomics studies have further demonstrated the feasibility of identifying the primary tumor site in brain metastases using quantitative feature extraction and classification models [20–22]. However, most existing studies have primarily focused on features extracted from the entire tumor region, while insufficiently considering the distinct characteristics of the peritumoral microenvironment in BM. This limitation may impede a deeper understanding of tumor biology and restrict further improvements in the accuracy (ACC) of BM origin prediction.

The brain–tumor interface (BTI) represents a dynamic transitional zone between tumor tissue and normal brain parenchyma, exhibiting distinct pathological characteristics that reflect tumor infiltration and provide critical information for distinguishing tumor subtypes [23–25]. The microenvironment of the BTI is substantially affected by the disruption of the blood–brain barrier (BBB) and the tumor-associated blood–tumor barrier (BTB), which highlights the heterogeneity of the tumor microenvironment [26]. Analysis of the BTI region may be of significant value in enabling the characterization of interactions between the tumor and adjacent tissues, and in providing valuable insights into the biological mechanisms and invasive behavior of BM [23, 24, 26, 27].

Previous studies have demonstrated that BTI regions of varying widths can capture substantial heterogeneity in the tumor microenvironment, thereby providing important insights into tumor invasion mechanisms [26, 28–30]. Although the value of BTI in predicting the origin of BM has been explored in prior work [23, 30], existing studies have primarily reported the potential relevance of the BTI region without systematically defining or comparing the spatial extent of the interface. Consequently, the optimal BTI width and its specific association with the primary origin of brain metastases remain insufficiently characterized. In this study, we therefore systematically evaluated and optimized the spatial definition of the BTI by comparing multiple interface widths (2, 4, 6, and 8 mm) within a unified radiomics framework. By directly assessing the diagnostic performance of radiomic features derived from different BTI extents and the whole tumor region across multiple classification tasks and validation cohorts, we aimed to clarify the contribution of BTI spatial configuration to origin prediction performance. Our findings provide exploratory evidence supporting the diagnostic relevance of BTI-derived radiomic features, while further prospective validation is required before clinical translation.

## Materials and methods

### Patients

This retrospective study was approved by the Medical Committee of Shengjing Hospital of China Medical University, the Ethics Committee of Liaoning Provincial Cancer Hospital (approval no: 20200819YG), and the Ethics Committee of Liaoning Provincial People’s Hospital (approval no: 2024K022). The inclusion of patients from 2020 to 2023 was covered under the same IRB approval. In accordance with the retrospective nature of the study and institutional regulations, the requirement for informed consent was waived by the ethics committee. A total of 462 patients from three clinical centers, who underwent pre-treatment T1-weighted contrast-enhanced (T1CE) and T2-weighted (T2W) brain MRI scans between January 2017 and December 2023, were included in the radiomics analysis. The inclusion criteria were as follows: (i) completion of pre-treatment T1CE and T2W brain MRI examinations; (ii) confirmation of the primary tumor site based on clinical history and imaging findings, with histopathological verification by biopsy. The exclusion criteria were: (i) incomplete clinical data; (ii) inadequate MRI image quality; and (iii) receipt of any prior treatment for brain metastases (e.g., radiotherapy, surgery, chemotherapy, or targeted therapy) before imaging acquisition.

**Fig. 1:**
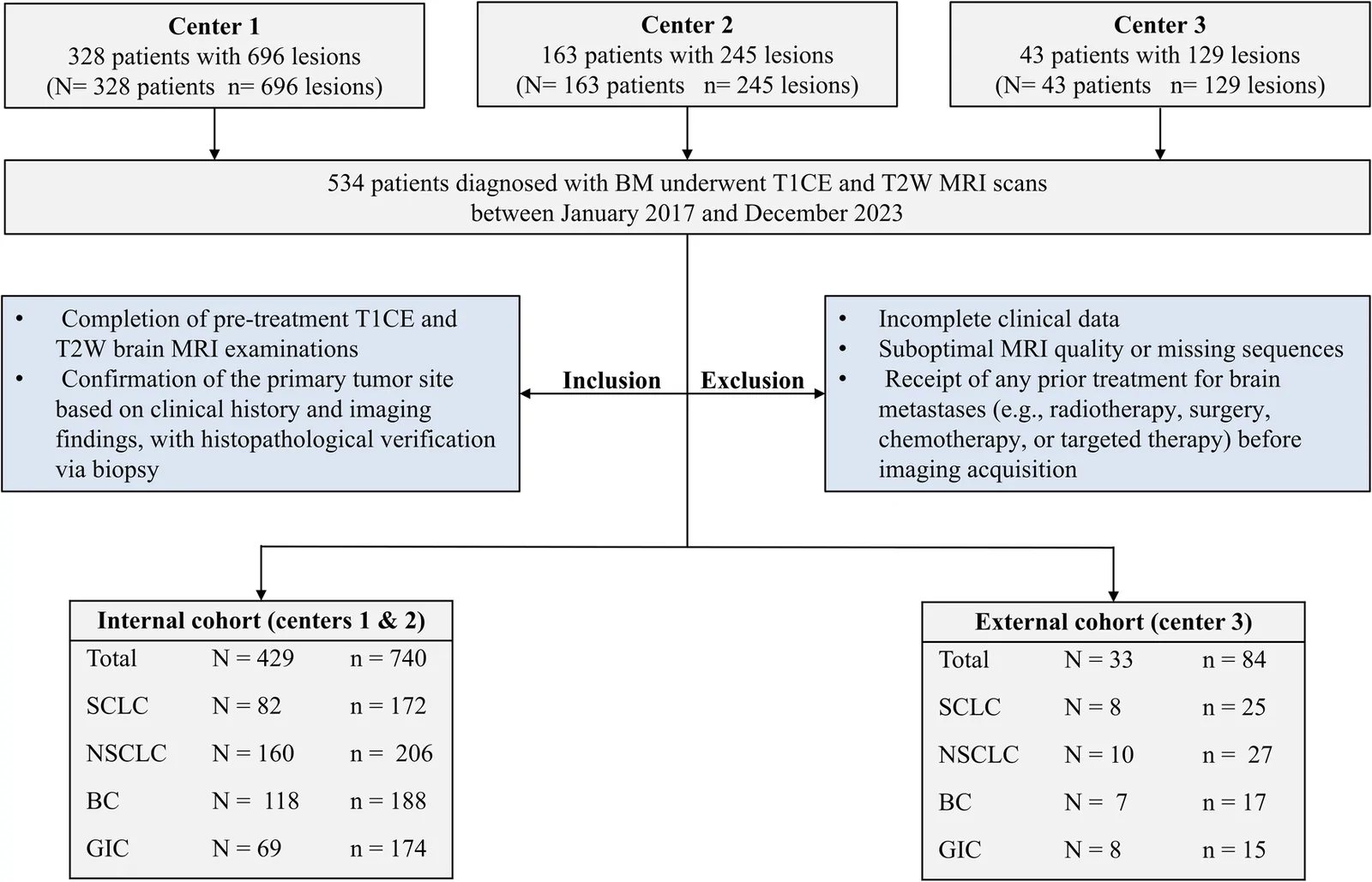
Patient selection flowchart showing the inclusion and exclusion process

In the primary cohort, a total of 429 patients from Center 1 and Center 2 were included, with 740 lesions analyzed. The primary tumor types of these lesions were NSCLC (160 patients, 206 lesions), SCLC (82 patients, 172 lesions), BC (118 patients, 188 lesions), and GIC (69 patients, 174 lesions). Patients were stratified and randomly assigned to the training and internal validation cohorts in a 2:1 ratio. As a result, the training cohort included 492 lesions (NSCLC = 137, SCLC = 115, BC = 124, GIC = 116) and the internal validation cohort included 248 lesions (NSCLC = 69, SCLC = 57, BC = 64, GIC = 58). Radiomics analyses were conducted at the lesion level, with each BM treated as one sample. Importantly, to avoid potential data leakage, all lesions from the same patient were assigned to the same cohort. For external validation, 84 lesions from 33 patients in Center 3 (NSCLC = 27, SCLC = 25, BC = 17, GIC = 15) were used as an independent test cohort to validate our findings. Fig. 1 presents the flowchart of patient inclusion and exclusion for this study.

### MRI acquisition

All patients underwent MRI scans using 3.0-T scanners across three centers. Specifically, imaging was performed on a Philips Ingenia system at Center 1, a Siemens Verio system at Center 2, and a General Electric Discovery system at Center 3. T1CE images were acquired following intravenous administration of gadolinium-diethylenetriamine pentaacetic acid (Gd-DTPA) at a dose of 0.2 mL/kg and an injection rate of 3 mL/s. A comprehensive summary of the MRI acquisition parameters is provided in Supplementary Appendix S1.

### Tumor segmentation and BTI generation

All BM lesions were manually annotated by two experienced radiologists under the supervision of a senior radiologist with 15 years of experience in brain MRI interpretation. Lesions and boundaries were delineated on T1CE and T2W images, slice by slice, using ITK-SNAP (version 3.6.0, www.itksnap.org). Annotation considered tumor morphology, contrast-enhanced boundaries, and relevant imaging features to ensure precision and consistency. To evaluate intra-observer reproducibility, one radiologist repeated the segmentation on the same randomly selected subset of cases after a two-week interval, blinded to the initial contours. BTI regions of 2, 4, 6, and 8 mm widths were generated in Python (version 3.9.13) by converting physical distances to pixel units, extracting BM lesion boundaries with skimage.measure, and dilating the boundaries both inward and outward to create corresponding ring-shaped BTI masks.

Inter- and intra-observer reproducibility of radiomic features was assessed using the intraclass correlation coefficient (ICC). Specifically, inter-observer ICC was calculated based on independent segmentations performed by the two radiologists, and intra-observer ICC was calculated using repeated segmentations by the same radiologist. Radiomic features with ICC > 0.85 were considered reproducible and retained for subsequent analysis. The inter- and intra-observer ICC values of the selected radiomic features are provided in Supplementary Tables S1–S3.

### Feature extraction and selection

Radiomic features were extracted from BM and BTI regions of varying widths (2, 4, 6, and 8 mm) using PyRadiomics (version 3.0.1) [31]. All preprocessing procedures were performed exclusively on the training set. In addition, feature selection and model parameter tuning were conducted using the training set only, and the trained models were applied unchanged to the internal validation and external test sets. For each T1CE and T2W sequence, 3934 features were extracted per region, including shape, first-order statistics, and texture. Additional features were obtained using various image filters. Further details are provided in Supplementary Appendix S1. To ensure feature reproducibility, all extracted radiomic features were evaluated using ICC analysis, and only features with ICC > 0.85 were retained for subsequent modeling. All statistical analyses were conducted using R software (version 4.2.2). Initially, 3146 reproducible features were retained following ICC filtering. After Z-score normalization, low-variance filtering, and pairwise correlation analysis (|r| > 0.9), the feature set was reduced to 512. Subsequently, the Mann–Whitney U test was performed to identify features with significant inter-group differences (*p* < 0.05). To ensure the robustness and reproducibility of the modeling process, we employed a stratified sampling strategy (2/3 training, 1/3 testing) via the sampling package. To obtain a stable and stratified cohort split, a random seed search was performed during the initial dataset partitioning stage. This procedure was only used to identify a training/testing split with balanced class distributions. After the final split was determined, all subsequent preprocessing, feature selection, and model construction procedures, including Wilcoxon filtering, least absolute shrinkage and selection operator (LASSO) regression with 10-fold cross-validation, and stepwise logistic regression, were conducted exclusively within the training cohort, while the testing cohort was used only for independent evaluation. All subsets of specific features have been documented in Supplementary Tables S1–S3 to facilitate independent verification.

### Construction of binary classification models

The radiomics signature (RS) was constructed as a linear combination of selected features and their coefficients. Binary logistic regression models were developed using features from BTI regions of varying widths (2, 4, 6, and 8 mm; BTI-RS-2 mm, BTI-RS-4 mm, BTI-RS-6 mm, BTI-RS-8 mm) and the entire tumor region (BM-RS). The BTI-derived RS models included classifiers for distinguishing lung cancer (LC) from non-lung cancer (NLC) (RS-BTI2-LC/NLC, RS-BTI4-LC/NLC, RS-BTI6-LC/NLC, RS-BTI8-LC/NLC), SCLC from NSCLC (RS-BTI2-SC/NSC, RS-BTI4-SC/NSC, RS-BTI6-SC/NSC, RS-BTI8-SC/NSC), and BC from GIC (RS-BTI2-BC/GIC, RS-BTI4-BC/GIC, RS-BTI6-BC/GIC, RS-BTI8-BC/GIC). Additionally, RS models based on features from the entire BM region were constructed to distinguish LC from NLC (RS-BM-LC/NLC), SCLC from NSCLC (RS-BM-SC/NSC), and BC from GIC (RS-BM-BC/GIC). To further assess the potential influence of inter-center imaging heterogeneity, we performed an additional sensitivity (SEN) analysis using ComBat harmonization. The detailed comparison results are presented in Table S6.

### Construction of multiclass classification models

To evaluate the ability of radiomics to identify among four types of BM concurrently, we adopted a modeling workflow consistent with the binary classification experiments. Standardized radiomic feature extraction was performed, followed by initial feature selection using the Wilcoxon rank-sum test (*p* < 0.05), and subsequent dimensionality reduction and key feature selection using LASSO regression. Subsequently, a multinomial logistic regression classifier was employed to construct the four-class model. Model training was performed using stratified cross-validation with multiple random seeds, and the performance of the four-class model was systematically evaluated.

### Interpretability analysis of the models

To enhance the interpretability of the RSs, this study employed SHAP (SHapley Additive exPlanations) [32, 33] to interpret the contribution of each selected radiomic feature. By computing and visualizing SHAP values for each RS, we compared the feature importance rankings and their contributions to the decision-making process. This analysis provides a foundation for understanding the role of the BTI region in predicting the origin of BM.

### Radiologist reading

Four radiologists with varying levels of experience (2, 10, 15, and 20 years) were invited to independently evaluate the patients with BM from the external test cohort. For each patient, the radiologists were provided with unprocessed T1CE and T2W MRI scans, along with clinical information including patient age, sex, and visually appreciable MRI-based morphological features of the BM (detailed in Table S5). The morphological features were observable and reflected conventional imaging criteria commonly used in routine radiological assessments.

Each radiologist independently determined the most likely primary site of the BM. Diagnostic performance was quantified using SEN and specificity (SPE), and a comparative analysis was conducted between the radiologists and the radiomics models to evaluate the relative diagnostic value of the models compared to radiologists.

### Statistical analysis

**Fig. 2:**
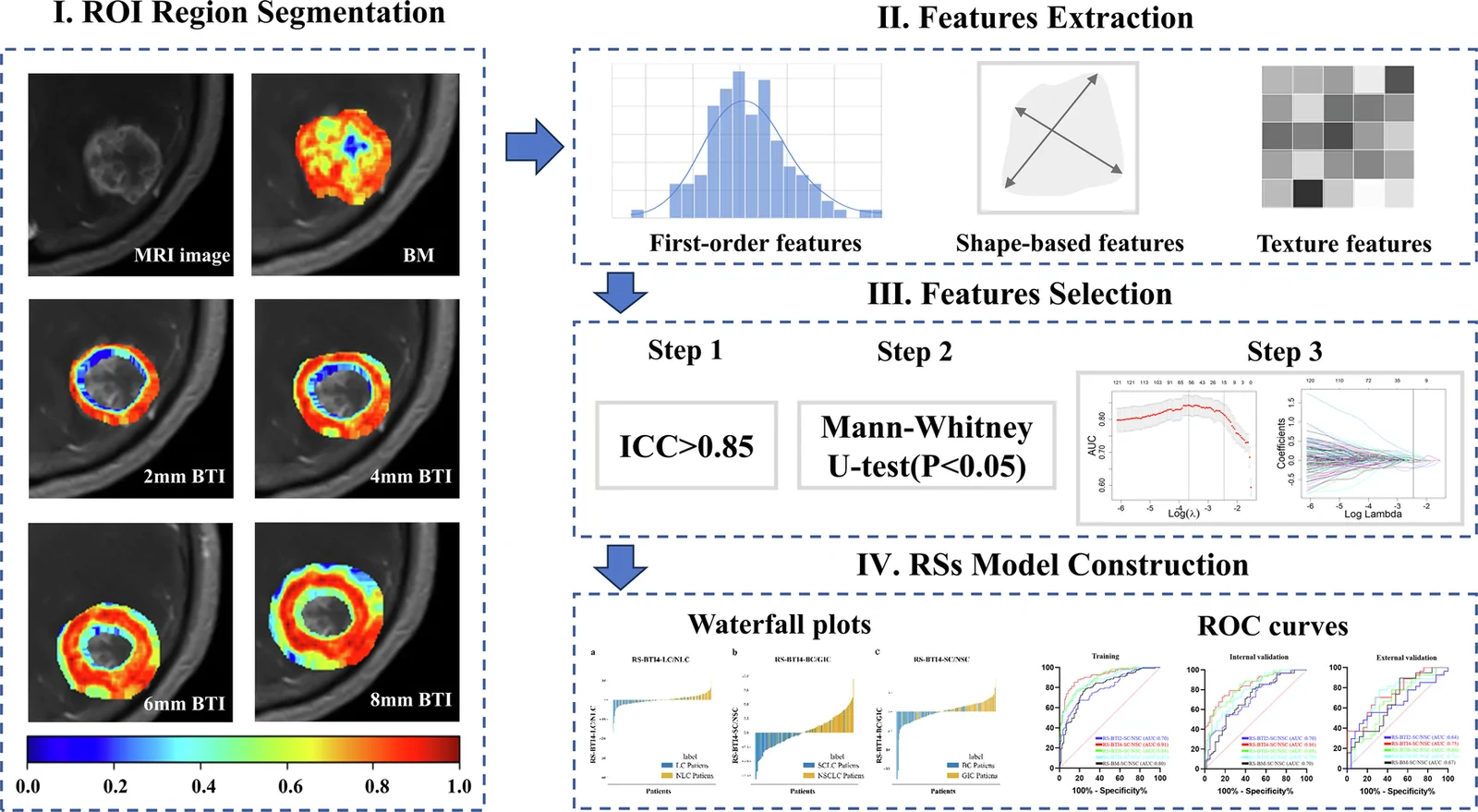
Workflow schematic of radiomics analyses. The region of interest region segmentation section displays an entropy map, where blue represents regions of low entropy (low information), and red represents regions of high entropy (high information)

All analyses were conducted using R (version 4.2.2). Statistical tests were two-sided, with *p* < 0.05 considered significant. Continuous variables were summarized as mean ± standard deviation and compared by *t*-test or Mann–Whitney *U*-test; categorical variables were reported as counts and percentages and compared using the chi-square test. Radiomics feature selection employed the Mann–Whitney *U*-test (*p* < 0.05). Model performance was assessed using receiver operating characteristic (ROC) curves, area under the curve (AUC), ACC, SEN, and SPE. The AUCs were reported with 95% confidence intervals (CI). Pairwise comparisons between models were performed using the DeLong test. To address within-patient non-independence, an additional patient-level SEN analysis was performed by retaining the largest lesion from each patient. Feature selection and logistic regression modeling were repeated using the patient-level training cohort, and the resulting models were evaluated in the internal and external validation cohorts. Figure 2 shows the study workflow.

## Results

### Patients characteristics

A total of 462 patients with BM were included in this study. Among them, 260 cases (56.3%) were of pulmonary origin, comprising 170 cases of NSCLC (36.8%) and 90 cases of SCLC (19.5%). The remaining 202 cases (43.7%) were of non-pulmonary origin, comprising 125 cases of BC (27.0%) and 77 cases of GIC (16.7%). Clinical characteristics of the patients are summarized in Table 1. There were no statistically significant differences in age or sex among the primary tumor groups (*p* > 0.05), suggesting that these variables have limited predictive value in determining the origin of BM. Overall, LC accounted for the largest proportion of brain metastases in the primary cohort, followed by BC and GIC. The distribution of tumor origins was comparable between the primary and external cohorts, supporting the representativeness of the external validation dataset. In addition, no significant differences in age or sex were observed among the primary tumor–origin groups, indicating a relatively balanced baseline clinical profile.

**Table 1:** Characteristics of patients with BM according to tumor origin in primary and external cohorts

Characteristic	Cohort	Tumor type		*p*	Tumor type		*p*	Tumor type		*p*
		LC	NLC		SCLC	NSCLC		BC	GIC	
Patients	Primary	242	187		82	160		118	69	
	External	18	15		8	10		7	8	
Lesions	Primary	378	362		172	206		188	174	
	External	52	32		25	27		17	15	
Ages (mean ± SD)	Primary	58.22 ± 8.13	57.02 ± 10.34	< 0.01	59.52 ± 8.71	57.55 ± 7.76	0.06	54.58 ± 9.95	61.2 ± 9.59	< 0.01
	External	63.00 ± 8.80	62.72 ± 12.65	0.87	60.76 ± 14.86	64.60 ± 3.78	0.86	62.10 ± 8.12	63.70 ± 18.10	0.85
Gender										
Male	Primary	132 (54.5%)	47 (25.1%)	0.05	58 (70.7%)	74 (46.3%)	0.07	0	47 (68.12%)	< 0.01
	External	8 (44.4%)	4 (26.6%)	0.13	3 (37.5%)	5 (50.0%)	0.96	0	4 (50.0%)	0.04
Female	Primary	110 (45.4%)	140 (74.9%)		24 (29.27%)	86 (53.7%)		118 (100%)	22 (32.83%)	
	External	10 (55.6%)	11 (73.4%)		5 (62.5%)	5 (50.0%)		7 (100.0%)	4 (50.0%)	

### Assessment of RSs based on BTI regions of varying widths and BM

**Fig. 3:**
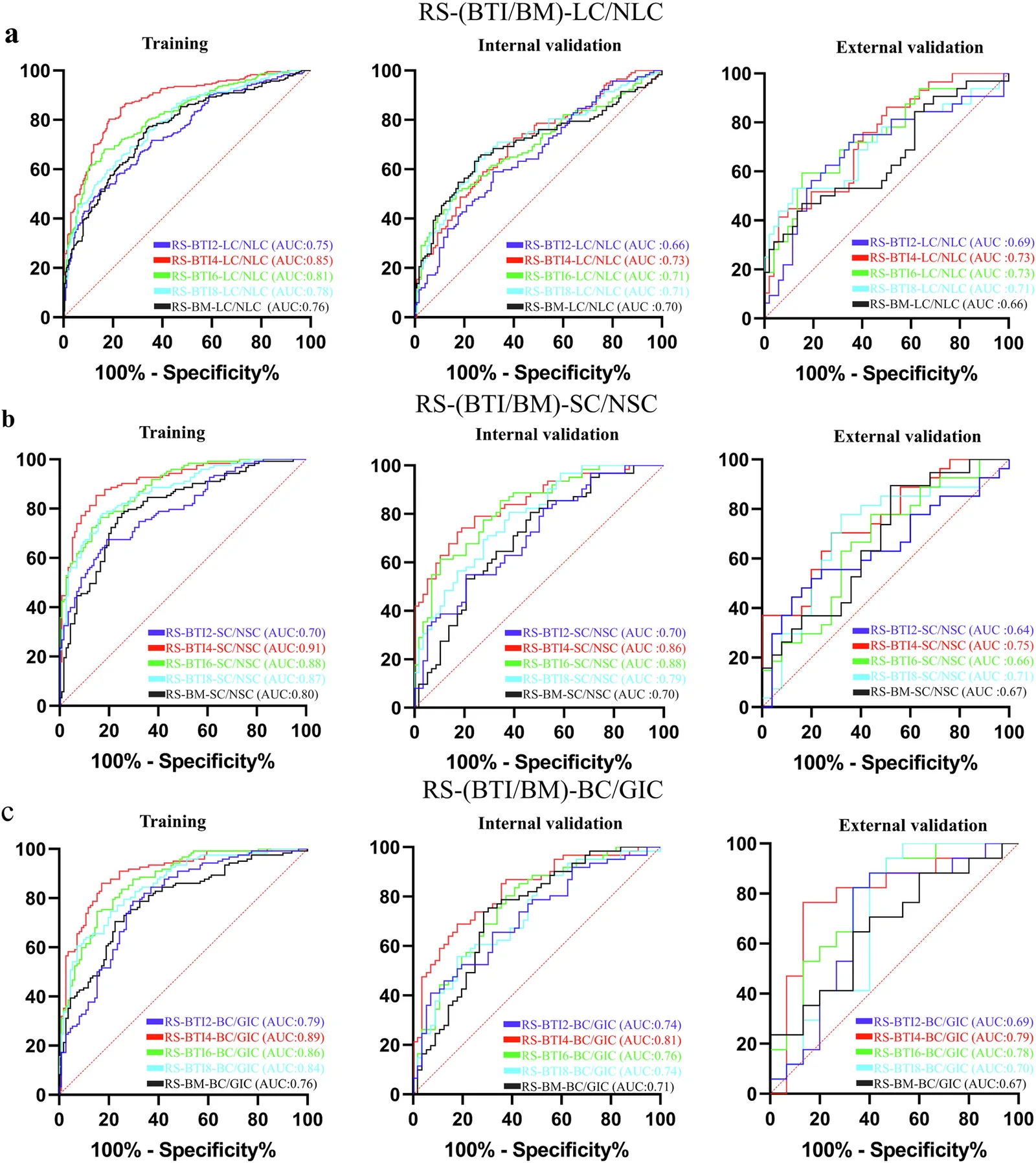
ROC curves of the developed RS-LC/NLC, RS-SC/NSC, and RS-BC/GIC models in the training (**a**), internal validation (**b**), and external validation (**c**) cohorts

To comprehensively evaluate the predictive value of BTI regions with varying widths, we constructed 15 RSs for three binary classification tasks: LC vs NLC, SCLC vs NSCLC, and BC vs GIC (Fig. 3a–c). For each task, RSs were developed using features derived from four BTI widths (2 mm, 4 mm, 6 mm, 8 mm), as well as from the entire tumor region. As summarized in Table 2 and detailed in Tables S1–S3, models based on BTI-4 mm features (RS-BTI4-LC/NLC, RS-BTI4-SC/NSC, and RS-BTI4-BC/GIC) consistently achieved the highest predictive performance across training, internal validation, and external validation cohorts. Model performance was evaluated using the AUC, with 95% CI reported for all models across the training, internal validation, and external validation cohorts. Pairwise comparisons between models were conducted using the DeLong test, and corresponding *p* values were consistently reported. Specifically, the AUCs of RS-BTI4-LC/NLC were 0.855, 0.726, and 0.739; those of RS-BTI4-SC/NSC were 0.910, 0.856, and 0.750; and those of RS-BTI4-BC/GIC were 0.890, 0.809, and 0.792, respectively. The ROC curves presented in Fig. 3 clearly illustrate the superior discriminative ability and stability of the BTI-4 mm models across different cohorts. Corresponding LASSO feature selection results are displayed in Fig. S1, while detailed RS formulae are provided in Appendix S2. Furthermore, DeLong test results in the external validation cohort showed that the BTI-4 mm model demonstrated significantly better performance than the BTI-2 mm model (*p* = 0.04) and the whole-tumor model (*p* = 0.02) for the SCLC vs NSCLC classification task, whereas the remaining pairwise comparisons in the external cohort did not reach statistical significance (Table S4). To account for within-patient correlation from multiple lesions, we performed an additional patient-level SEN analysis. Compared with the original lesion-level analysis, changes in patient-level AUC ranged from −0.063 to +0.026, with the overall discriminatory pattern generally preserved. In the external validation cohort, AUC changes were +0.005, −0.017, and −0.056 for LC vs NLC, SCLC vs NSCLC, and BC vs GIC, respectively. These findings suggest that the main conclusions were not substantially driven by within-patient lesion correlation (Table S7).

**Table 2:** The predictive performance of RS models based on BTI of varying widths and the BM regions

	Training cohort				Internal validation cohort				External cohort			
RS	AUC (95% CI)	ACC	SEN	SPE	AUC (95% CI)	ACC	SEN	SPE	AUC (95% CI)	ACC	SEN	SPE
RS-BTI2-LC/NLC	0.746 (0.703–0.790)	0.675	0.899	0.472	0.665 (0.596–0.734)	0.637	0.825	0.470	0.692 (0.569–0.816)	0.643	0.635	0.75
RS-BTI4-LC/NLC	0.855 (0.821–0.889)	0.779	0.786	0.803	0.726 (0.662–0.789)	0.667	0.600	0.752	0.739 (0.629–0.850)	0.691	0.5	0.862
RS-BTI6-LC/NLC	0.807 (0.768–0.846)	0.724	0.815	0.687	0.708 (0.642–0.776)	0.705	0.842	0.564	0.739 (0.627–0.852)	0.667	0.846	0.594
RS-BTI8-LC/NLC	0.784 (0.744–0.825)	0.694	0.584	0.841	0.714 (0.648–0.780)	0.709	0.842	0.581	0.710 (0.587–0.838)	0.738	0.885	0.531
RS-BM-LC/NLC	0.762 (0.719–0.805)	0.679	0.676	0.742	0.699 (0.630–0.767)	0.671	0.742	0.632	0.659 (0.534–0.787)	0.649	0.846	0.531
RS-BTI2-SC/NSC	0.701 (0.739–0.850)	0.710	0.843	0.634	0.705 (0.612–0.797)	0.650	0.793	0.532	0.643 (0.489–0.798)	0.616	0.88	0.556
RS-BTI4-SC/NSC	0.910 (0.874–0.947)	0.832	0.878	0.821	0.856 (0.791–0.921)	0.767	0.845	0.742	0.750 (0.618–0.881)	0.635	0.72	0.704
RS-BTI6-SC/NSC	0.89 (0.846–0.926)	0.794	0.861	0.740	0.88 (0.755–0.899)	0.717	0.707	0.806	0.664 (0.513–0.814)	0.654	0.56	0.778
RS-BTI8-SC/NSC	0.867 (0.823–0.911)	0.769	0.896	0.699	0.789 (0.710–0.868)	0.658	0.897	0.548	0.711 (0.564–0.858)	0.519	0.68	0.778
RS-BM-SC/NSC	0.805 (0.749–0.861)	0.697	0.765	0.764	0.701 (0.607–0.796)	0.642	0.534	0.806	0.670 (0.508–0.831)	0.614	0.52	0.895
RS-BTI2-BC/GIC	0.779 (0.719–0.839)	0.665	0.676	0.861	0.709 (0.616–0.801)	0.650	0.857	0.475	0.694 (0.494–0.894)	0.594	0.667	0.824
RS-BTI4-BC/GIC	0.890 (0.848–0.932)	0.811	0.829	0.820	0.809 (0.730–0.888)	0.744	0.643	0.869	0.792 (0.618–0.966)	0.686	0.867	0.768
RS-BTI6-BC/GIC	0.864 (0.818–0.910)	0.755	0.730	0.844	0.745 (0.656–0.835)	0.658	0.643	0.738	0.780 (0.613–0.948)	0.656	0.667	0.823
RS-BTI8-BC/GIC	0.838 (0.789–0.888)	0.742	0.856	0.648	0.740 (0.650–0.830)	0.667	0.839	0.525	0.702 (0.501–0.903)	0.719	0.600	0.882
RS-BM-BC/GIC	0.786 (0.728–0.844)	0.670	0.775	0.705	0.737 (0.646–0.829)	0.658	0.714	0.738	0.667 (0.474–0.860)	0.656	0.667	0.647

We also performed calibration curve analysis and decision curve analysis (DCA) for RS-BTI4 models. Calibration curves (Fig. S2) showed strong agreement between predicted and observed outcomes, indicating good model calibration. DCA further demonstrated that the 4-mm BTI models provided the highest net clinical benefit across threshold probabilities, confirming superior clinical utility and applicability.

### Assessment of multiclass classification model

Table 3 summarizes the predictive performance of the multiclass models using BTI regions of varying widths and the BM region. The 4-mm BTI model achieved the highest AUC in training but showed a significant decline in validation performance, indicating potential overfitting and limited generalizability. To mitigate this, strategies such as LASSO regularization, feature reproducibility filtering, and stratified k-fold cross-validation were applied. In contrast, binary classification models demonstrated superior AUC, stability, and more balanced SEN and SPE, likely due to clearer class boundaries and reduced class imbalance.

**Table 3:** The predictive performance of multiclass classification models based on BTI of varying widths and the BM regions

		Training cohort				Validation cohort			
RS	Tumor Type	AUC	ACC	SPE	SEN	AUC	ACC	SPE	SEN
RS_BTI2	SCLC	0.831	0.640	0.643	0.923	0.761	0.516	0.540	0.903
	NSCLC	0.792	0.505	0.562	0.901	0.673	0.703	0.589	0.689
	BC	0.731	0.622	0.661	0.703	0.620	0.607	0.808	0.589
	GIC	0.751	0.559	0.742	0.658	0.628	0.625	0.656	0.875
RS_BTI4	SCLC	0.885	0.703	0.739	0.901	0.660	0.532	0.699	0.581
	NSCLC	0.878	0.667	0.756	0.820	0.625	0.689	0.616	0.892
	BC	0.838	0.559	0.760	0.766	0.566	0.643	0.663	0.500
	GIC	0.849	0.640	0.577	0.946	0.669	0.736	0.641	0.681
RS_BTI6	SCLC	0.878	0.568	0.853	0.739	0.739	0.565	0.827	0.532
	NSCLC	0.805	0.586	0.697	0.766	0.643	0.568	0.516	0.716
	BC	0.800	0.514	0.751	0.730	0.670	0.571	0.659	0.643
	GIC	0.812	0.613	0.784	0.685	0.634	0.583	0.823	0.569
RS_BTI8	SCLC	0.830	0.586	0.745	0.768	0.685	0.613	0.743	0.597
	NSCLC	0.826	0.586	0.724	0.793	0.662	0.695	0.658	0.662
	BC	0.787	0.523	0.580	0.829	0.671	0.625	0.779	0.500
	GIC	0.795	0.667	0.814	0.667	0.644	0.697	0.531	0.708
RS_BM	SCLC	0.780	0.523	0.775	0.739	0.791	0.661	0.693	0.774
	NSCLC	0.838	0.549	0.709	0.726	0.707	0.527	0.732	0.608
	BC	0.773	0.577	0.658	0.811	0.638	0.607	0.726	0.554
	GIC	0.746	0.568	0.595	0.856	0.691	0.556	0.609	0.736

### Feature importance and discriminative patterns of RS-BTI4 models

**Fig. 4:**
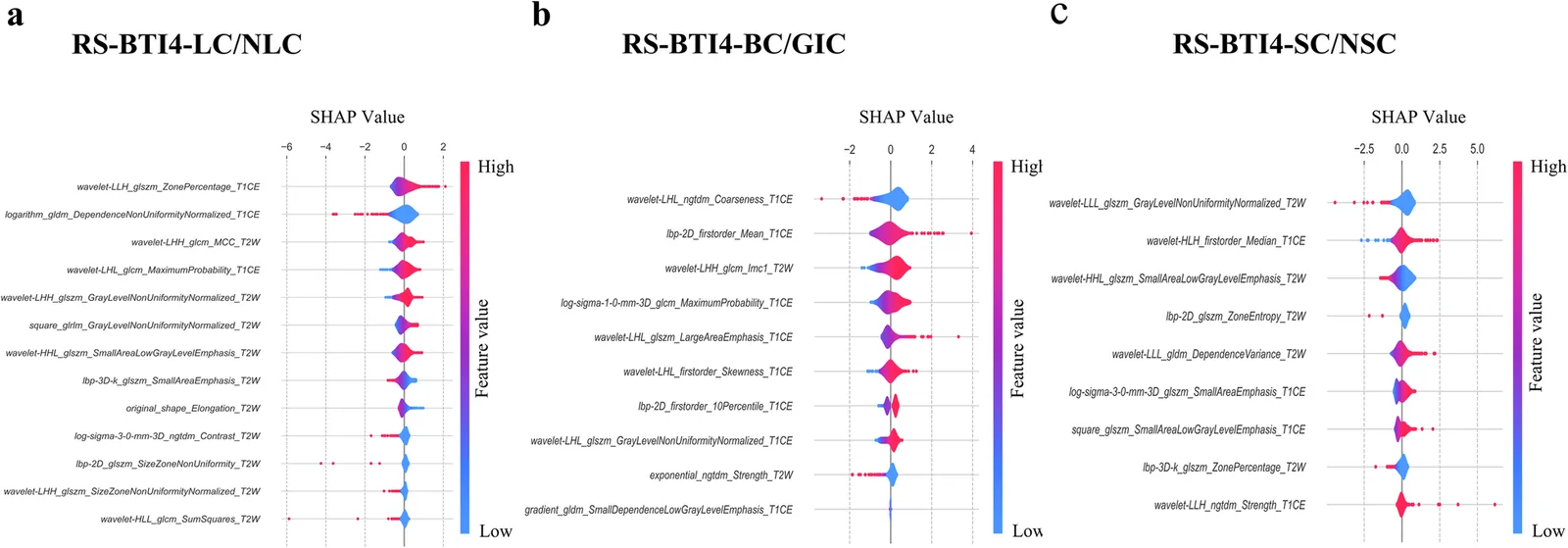
SHAP-based feature importance analysis of the RS-BTI4 models for (**a**) LC vs. NLC, (**b**) BC vs. GIC, and (**c**) SCLC vs. NSCLC, the color in the figure indicates the direction and magnitude of each feature’s impact on class discrimination, facilitating intuitive understanding of the model’s decision-making process

Figure 4 presents the SHAP-based feature importance of the three RS-BTI4 models in the training cohort. Each model identified distinct texture-derived radiomic features as key contributors to tumor origin classification. The color scale indicates both the direction and magnitude of feature impact, illustrating the heterogeneity captured within the 4-mm BTI and the diagnostic relevance of multimodal MR texture and morphology [20, 30, 34].

Notably, the most influential features were derived from the gray-level co-occurrence matrix (GLCM), gray-level size zone matrix (GLSZM), and gray-level dependence matrix (GLDM), reflecting heterogeneity and microstructural complexity within the 4-mm BTI. Features such as GLSZM nonuniformity and GLDM dependence variance corresponded to irregular signal distribution and peritumoral intensity variation, radiological correlates of infiltrative margins, vascular remodeling, and edema typically observed in lung-derived metastases. Conversely, GLCM maximum probability and large area emphasis, which dominated in breast- and gastrointestinal-derived metastases, represented homogeneous enhancement and compact morphology, consistent with denser cellularity and perivascular infiltration on histopathology. These associations substantiate the biological interpretability of the RS-BTI4 models, showing that radiomic features quantitatively capture imaging-pathology concordant patterns related to tumor–microenvironment interactions, including blood–brain barrier (BBB) disruption, angiogenesis, and reactive gliosis [35, 36].

**Fig. 5:**
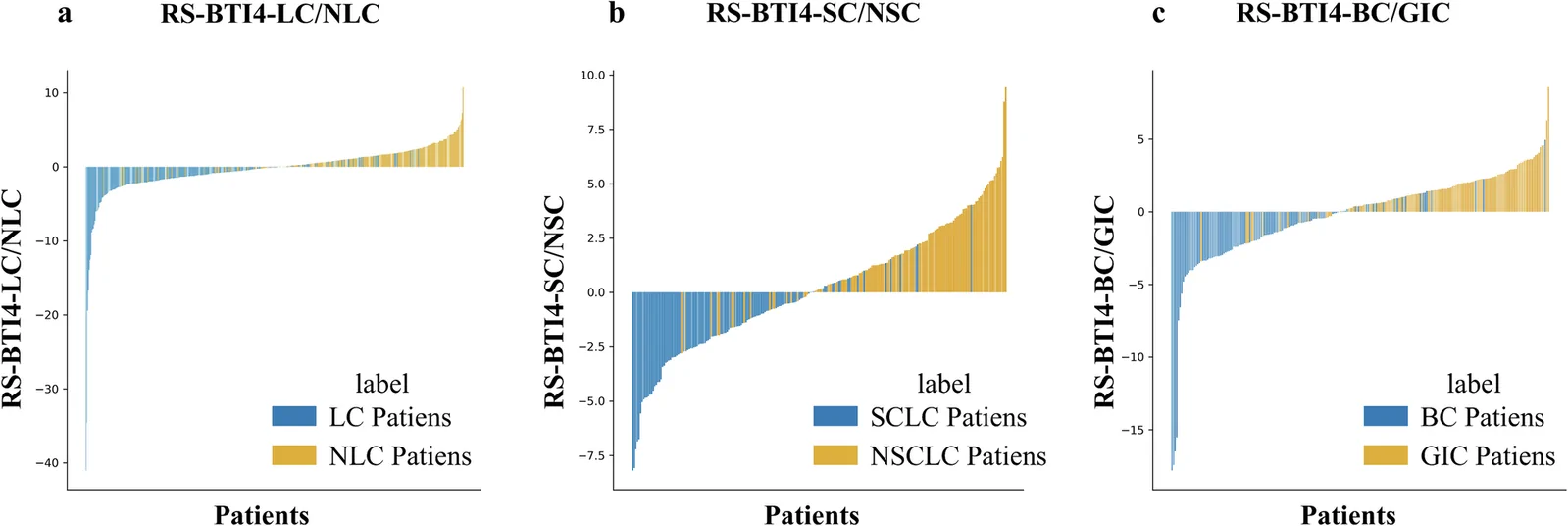
Distribution of individual prediction scores for the RS-BTI4 models: (**a**) LC vs. NLC, (**b**) SCLC vs. NSCLC, and (**c**) BC vs. GIC. For each model, the prediction scores are arranged in ascending order, with different colors indicating the primary tumor categories

The 4-mm BTI model achieved superior discriminative performance compared with whole-tumor and other BTI-width models, effectively capturing biologically relevant interface information while suppressing background noise [28, 30, 34]. Figure 5 shows clearly separated prediction score distributions with minimal overlap, confirming the RS-BTI4 models’ robustness and reliability in distinguishing brain metastases of different primary origins.

### Radiologist reading

The results comparing radiologist interpretations and radiomics model performance are summarized in Table 4. The four radiologists participating in this study were board-certified and subspecialized in neuroradiology, with 2, 10, 15, and 20 years of experience in neuro-oncologic imaging, respectively. Their diagnostic performance showed moderate ACC with noticeable inter-observer variability. In contrast, the RS-BTI4 model demonstrated superior ACC and consistency across all classification tasks, significantly outperforming human readers. These findings suggest that the BTI-based radiomics framework not only surpasses expert-level interpretation but also provides a more objective and reproducible tool for identifying the primary origin of brain metastases.

**Table 4:** Comparison of radiologist assessment and radiomics-based identification of metastatic types of BM

Tumor type	Reader 1		Reader 2		Reader 3		Reader 4		RS-BTI4 model	
	SPE	SEN	SPE	SEN	SPE	SEN	SPE	SEN	SPE	SEN
LC/NLC	0.625	0.534	0.615	0.532	0.654	0.523	0.564	0.513	0.685	0.771
SC/NSC	0.631	0.545	0.563	0.543	0.538	0.620	0.524	0.524	0.872	0.764
BC/GIC	0.675	0.672	0.613	0.587	0.737	0.650	0.572	0.620	0.898	0.786

## Discussion

The BTI is dynamically associated with angiogenesis, tumor cell migration, and immune suppression, yielding high imaging information density and reflecting complex tumor–host interactions [23, 26, 30, 35]. Although BTI-based radiomic analysis is underexplored in BM, this study systematically constructed classification models based on BTI regions. Diagnostic performance of BTI-derived features was assessed across LC vs NLC, SCLC vs NSCLC, and BC vs GIC tasks. Models utilizing BTI features consistently outperformed those based on the BM region, achieving higher AUCs and improved stability in both training and validation cohorts. These findings suggest that the BTI is not merely an anatomic boundary, but a biologically active region that contains origin-related information complementary to, and partly independent of, conventional whole-tumor radiomic features. Therefore, the present study further clarifies the added value of BTI-based radiomics for BM origin prediction.

To our knowledge, this study is the first to systematically compare BTI regions of varying widths (2, 4, 6, and 8 mm) and the entire BM region across multiple classification tasks. The 4-mm BTI model consistently achieved the highest AUCs for RS-BTI4-SCLC/NSCLC (0.910/0.856), RS-BTI4-BC/GIC (0.890/0.809), and RS-BTI4-LC/NLC (0.855/0.726), significantly outperforming all other BTI widths and the BM region. We speculate that the 4-mm BTI region aligns with the clinical “biological boundary,” where tumor infiltration, angiogenesis, and immune cell activity are most prominent [35–38]. This zone encompasses the tumor microenvironment, reactive gliosis, and local inflammation, enabling BTI-derived radiomic features to capture relevant tissue heterogeneity with minimal background noise. Regions that are too narrow may miss key pathological changes, while excessively broad regions can dilute discriminative power by including normal brain tissue. Consistently, Hu et al [39] found that texture heterogeneity within a 2–5 mm bandwidth is closely linked to tumor margin biology, underscoring the diagnostic value of the 4-mm BTI region.

Different primary tumors exhibit distinct infiltration patterns within brain tissue. LC typically displays an expansive growth pattern with well-defined imaging borders [40]; BC often demonstrates perivascular diffuse infiltration, resulting in indistinct margins; while GIC is characterized by irregular morphology and pronounced heterogeneity [41]. These histological and biological differences can be effectively captured by the radiomic features extracted from the BTI region. The findings of this study indicate that high-dimensional features, such as texture and morphology, extracted from the BTI region possess greater discriminative power for identifying the primary origin, further supporting the notion that the BTI region provides greater diagnostic value than the BM region features, as suggested by Jiang et al [30]. Meanwhile, Boire et al [2] have noted that the adaptive mechanisms of brain metastases involve disruption of the BBB, remodeling of the immune microenvironment, and reactive gliosis, all of which are likely to manifest as diagnostically relevant imaging patterns in the texture features of the BTI region. Our study directly compared multiple BTI widths with whole-tumor radiomics under the same modeling framework, confirming BTI-specific added value. ComBat SEN analysis further showed comparable performance before and after harmonization, suggesting robustness to center-related variability.

The added value of the BTI approach lies in isolating the active tumor–host interface while reducing nonspecific intratumoral noise. Whole-tumor radiomics includes core changes such as necrosis, cystic degeneration, and hemorrhage, which are common across advanced brain metastases and may dilute origin-specific signals. By excluding the core and focusing on the 4-mm BTI, our method improves the signal-to-noise ratio and enhances predictive stability, particularly in external validation.

By selecting radiomic features from the 4-mm BTI region, this study identified 13, 10, and 9 discriminative features for the RS-BTI4-LC/NLC, RS-BTI4-SC/NSC, and RS-BTI4-BC/GIC models, respectively, of which 84% (27/32) were texture-based. SHAP analysis demonstrated that texture features derived from the GLCM, GLSZM, and GLDM contributed most strongly to model discrimination. These features quantify spatial gray-level correlations, homogeneous intensity zone distributions, and gray-level dependence, capturing pronounced signal heterogeneity and intensity variation at the tumor margin on multiparametric MRI. Such imaging characteristics reflect key pathological processes at the BTI, including infiltrative tumor margins, heterogeneous angiogenesis, BBB disruption, perivascular infiltration, reactive gliosis, and peritumoral edema. Importantly, these processes differ across primary tumor types and are manifested as distinct growth patterns and microenvironmental remodeling. Consistent with known histopathological behaviors, GLCM-based features were more closely associated with compact morphology and relative cellular homogeneity typically observed in breast and GIC metastases, whereas GLSZM- and GLDM-based features reflected irregular margins, vascular remodeling, and increased heterogeneity characteristic of LC metastases [23, 30, 34, 35, 42–44]. Collectively, these findings indicate that the 4-mm BTI region provides an optimal spatial context for capturing biologically meaningful grayscale heterogeneity, serving as a robust imaging surrogate of tumor–microenvironment interactions and explaining the superior discriminative performance of BTI-based radiomics models.

In addition to its diagnostic potential, BTI-derived radiomic features offer promising applications in clinical decision-making, particularly for patients with brain metastasis of unknown primary (CUP-BM). Conventional MRI often struggles to provide specific identification of the primary tumor origin, but the radiomic features derived from the BTI could serve as valuable noninvasive biomarkers, helping to guide diagnosis and treatment [13, 30]. These features reflect underlying biological processes, such as vascular remodeling, immune cell infiltration, and invasive growth patterns, which could offer insights into tumor biology and facilitate patient stratification [28, 34]. BTI-based radiomics could be used after routine MRI and lesion segmentation to provide a probability estimate of the likely primary origin, complementing rather than replacing pathology or systemic evaluation. In CUP-BM, such predictions may help prioritize targeted tests, such as thoracic CT or breast MRI, reducing diagnostic burden and time. However, as CUP-BM patients were not validated here, this remains a future application. The observed 0.10–0.15 reduction in AUC during external validation, relative to training performance, reveals a susceptibility to center-specific imaging characteristics and potential overfitting. To mitigate such inter-center heterogeneity, future efforts should prioritize the integration of expansive multicenter cohorts alongside advanced harmonization frameworks to ensure broader clinical applicability.

This study has several limitations. First, although the 4-mm BTI region demonstrated optimal performance in our dataset, its universal applicability across different primary tumor types, tumor volumes, MRI parameters, and growth patterns remains uncertain. Second, the primary analysis was conducted at the lesion level and may therefore be affected by within-patient correlation when multiple lesions are present. Although the patient-level SEN analysis showed that the overall discriminatory pattern was generally preserved across the training, internal validation, and external validation cohorts, this analysis does not fully account for the hierarchical structure of multiple lesions within individual patients. Larger studies using hierarchical or generalized estimating equation approaches are therefore warranted. Moreover, only basic demographic variables (age and sex) were analyzed, whereas other potentially relevant clinical variables (e.g., systemic treatment history, molecular subtype, and extracranial disease burden) were not uniformly available across centers and were therefore not incorporated into the analysis. Finally, the proposed models have not yet been validated in patients with BM from an unknown primary (CUP-BM), an essential clinical subgroup accounting for approximately 2–15% of BM cases [13]. Considering the clinical significance and diagnostic challenges of CUP-BM, future research could focus on developing BTI-based fingerprinting strategies incorporating individual imaging features to facilitate more precise source tracking in this population.

## Conclusion

Radiomics features from the BTI, particularly within a 4-mm zone, provide a discriminative tool for identifying the primary origin of brain metastases by capturing unique microenvironment interactions. In this study, the 4-mm BTI-based model showed the most favorable performance among different BTI widths and outperformed conventional whole-tumor radiomics models across the main classification tasks, highlighting the added value of interface-focused radiomic analysis. Although the small external cohort and potential scanner-related variability limit current generalizability, this approach offers a promising basis for clinical diagnostic support and may provide a noninvasive adjunct for prioritizing the diagnostic workup in patients with uncertain primary origin. Future work should focus on large-scale prospective validation and automated frameworks to refine its application, especially for CUP-BM.

## Supplementary information

ELECTRONIC SUPPLEMENTARY MATERIAL

## Data Availability

The datasets generated and/or analyzed during the current study are not publicly available due to restrictions but are available from the corresponding author upon reasonable request.

## Abbreviations

ACC: Accuracy
AUC: Area under the ROC curve
BBB: Blood–brain barrier
BC: Breast cancer
BM: Brain metastasis
BTI: Brain–tumor interface
CI: Confidence interval
CT: Computed tomography
DCA: Decision curve analysis
GIC: Gastrointestinal cancer
GLCM: Gray-level co-occurrence matrix
GLDM: Gray-level dependence matrix
GLSZM: Gray-level size zone matrix
ICC: Intraclass correlation coefficient
LASSO: Least absolute shrinkage and selection operator
LC: Lung cancer
MRI: Magnetic resonance imaging
NLC: Non-lung cancer
NSCLC: Non-small cell lung cancer
ROC: Receiver operating characteristic
RS: Radiomics signature
SCLC: Small cell lung cancer
SEN: Sensitivity
SHAP: Shapley additive explanations
SPE: Specificity
T1CE: Contrast-enhanced T1 weighted
T2W: T2-weighted
