# Supplementary material for: Radiomics analysis of brain–tumor interface on multiparametric MRI for prediction of brain metastasis primary site
Source: Insights Imaging. 2026 Nov 6;17:286. doi: 10.1186/s13244-026-02464-5 (PMC13743695; doi:10.1186/s13244-026-02464-5)
Supplement: Supplementary file 1 [file 13244_2026_2464_MOESM1_ESM.pdf]

# Radiomics Analysis of Brain-Tumor Interface on Multiparametric MRI for Prediction of Brain Metastasis Primary Site

## ELECTRONIC SUPPLEMENTARY MATERIAL

Insights Imaging (2026) Jiang M, Han B, Huang Y, et al.

**Table S1. Performance of Selected Features by RS-BTI 4-LC/NLC in Differentiating LC and NLC**

| Feature | Sequence | Mean±SD | | AUC | *P* | ICC (inter-) | ICC (intra-) |
|---|---|---|---|---|---|---|---|
| | | LC | NLC | | | | |
| logarithm_gldm_DependenceNonUniformityNormalized | T1CE | 0.13±0.06 | 0.12±0.03 | 0.558 | 0.028 | 0.981 | 0.972 |
| wavelet-LHL_glcm_MaximumProbability | T1CE | 0.36±0.07 | 0.37±0.06 | 0.559 | 0.028 | 0.980 | 0.959 |
| wavelet-LLH_glszm_ZonePercentage | T1CE | 0.11±0.09 | 0.13±0.09 | 0.554 | 0.043 | 0.901 | 0.929 |
| lbp-2D_glszm_SizeZoneNonUniformity | T2W | 1.02±0.15 | 1±0 | 0.511 | 0.044 | 0.966 | 0.971 |
| lbp-3D-k_glszm_SmallAreaEmphasis | T2W | 0.35±0.15 | 0.3±0.15 | 0.585 | <0.001* | 0.955 | 0.982 |
| log-sigma-3-0-mm-3D_ngtdm_Contrast | T2W | 0.02±0.02 | 0.02±0.01 | 0.577 | 0.003 | 0.954 | 0.939 |
| original_shape_Elongation_T2W | T2W | 0.81±0.15 | 0.77±0.17 | 0.560 | 0.032 | 0.991 | 0.975 |
| square_glrlm_GrayLevelNonUniformityNormalized | T2W | 0.41±0.23 | 0.45±0.23 | 0.991 | <0.001* | 0.966 | 0.958 |
| wavelet-HHL_glszm_SmallAreaLowGrayLevelEmphasis | T2W | 0.19±0.1 | 0.22±0.1 | 0.567 | <0.001* | 0.968 | 0.991 |
| wavelet-HLL_glcm_SumSquares | T2W | 0.32±0.35 | 0.28±0.07 | 0.553 | <0.001* | 0.981 | 0.972 |
| wavelet-LHH_glcm_MCC | T2W | 0.35±0.1 | 0.37±0.1 | 0.565 | <0.001* | 0.963 | 0.982 |
| wavelet-LHH_glszm_GrayLevelNonUniformityNormalized | T2W | 0.44±0.1 | 0.46±0.08 | 0.566 | 0.009 | 0.944 | 0.969 |
| wavelet-LHH_glszm_SizeZoneNonUniformityNormalized | T2W | 0.09±0.06 | 0.08±0.04 | 0.569 | 0.01 | 0.972 | 0.979 |

**Table S2. Performance of Selected Features by RS-BTI 4-SC/NSC in Differentiating SCLC and NSCLC**

| Feature | Sequence | Mean±SD | | AUC | *P* | ICC (inter-) | ICC (intra-) |
|---|---|---|---|---|---|---|---|
| | | SCLC | NSCLC | | | | |
| gradient_gldm_SmallDependenceLowGrayLevelEmphasis | T1CE | 0.03±0.01 | 0.02±0.01 | 0.7493 | <0.001* | 0.983 | 0.969 |
| lbp-2D_firstorder_10Percentile | T1CE | 2.27±0.62 | 2.64±0.52 | 0.6611 | <0.001* | 0.977 | 0.952 |
| lbp-2D_firstorder_Mean | T1CE | 4.39±0.17 | 4.54±0.28 | 0.6711 | <0.001* | 0.872 | 0.939 |
| log-sigma-1-0-mm-3D_glcm_MaximumProbability | T1CE | 0.45±0.17 | 0.58±0.17 | 0.6921 | <0.001* | 0.953 | 0.989 |
| wavelet-LHL_firstorder_Skewness_T1CE | T1CE | -0.18±0.73 | 0.16±0.74 | 0.6505 | <0.001* | 0.967 | 0.992 |
| wavelet-LHL_glszm_GrayLevelNonUniformityNormalized | T1CE | 0.4±0.13 | 0.48±0.1 | 0.6638 | <0.001* | 0.959 | 0.947 |
| wavelet-LHL_glszm_LargeAreaEmphasis | T1CE | 0.06±0.09 | 0.02±0.04 | 0.7749 | <0.001* | 0.958 | 0.955 |
| exponential_ngtdm_Strength | T2W | 0.19±0.39 | 0.05±0.15 | 0.577 | <0.001* | 0.991 | 0.987 |
| wavelet-LHH_glcm_Imc1 | T2W | -0.13±0.05 | -0.09±0.04 | 0.7413 | <0.001* | 0.970 | 0.979 |
| wavelet-LHL_ngtdm_Coarseness | T1CE | 0.01±0.01 | 0.01±0.01 | 0.7089 | <0.001* | 0.988 | 0.982 |

**Table S3. Performance of Selected Features by RS-BTI 4-BC/GIC in Differentiating BC and GIC**

| Feature | Sequence | Mean±SD | | AUC | *P* | ICC (inter-) | ICC (intra-) |
|---|---|---|---|---|---|---|---|
| | | BC | GIC | | | | |
| log-sigma-3-0-mm-3D_glszm_SmallAreaEmphasis | T1CE | 0.12±0.12 | 0.18±0.13 | 0.6402 | <0.001* | 0.971 | 0.978 |
| square_glszm_SmallAreaLowGrayLevelEmphasis | T1CE | 0.04±0.04 | 0.06±0.06 | 0.629 | 0.001 | 0.982 | 0.992 |
| wavelet-HLH_firstorder_Median | T1CE | -0.01±0.3 | 0.12±0.31 | 0.625 | 0.001 | 0.937 | 0.946 |
| wavelet-LLH_ngtdm_Strength | T1CE | 1.36±1.98 | 2.83±6.83 | 0.596 | 0.03 | 0.959 | 0.972 |
| lbp-2D_glszm_ZoneEntropy | T2W | 0.21±0.45 | 0.05±0.22 | 0.570 | <0.001* | 0.988 | 0.993 |
| lbp-3D-k_glszm_ZonePercentage | T2W | 0.01±0.01 | 0.01±0 | 0.576 | 0.027 | 0.973 | 0.988 |
| wavelet-HHL_glszm_SmallAreaLowGrayLevelEmphasis | T2W | 0.22±0.1 | 0.2±0.1 | 0.581 | 0.032 | 0.946 | 0.961 |
| wavelet-LLL_gldm_DependenceVariance | T2W | 4.22±1.23 | 4.66±1.65 | 0.578 | 0.023 | 0.969 | 0.943 |
| wavelet-LLL_glszm_GrayLevelNonUniformityNormalized | T2W | 0.09±0.06 | 0.07±0.03 | 0.599 | 0.002 | 0.985 | 0.951 |

**Table S4: DeLong test results of LC vs. NLC, SCLC vs. NSCLC and BC vs. GIC -originated BM, 4mm BTI**

| RS | Training cohort | | | | | Internal validation cohort | | | | | External cohort | | | | |
|---|---|---|---|---|---|---|---|---|---|---|---|---|---|---|---|
| | AUC (95% CI) | ACC | SEN | SPE | *P* | AUC (95% CI) | ACC | SEN | SPE | *P* | AUC (95% CI) | ACC | SEN | SPE | *P* |
| RS-BTI2-LC/NLC | 0.746 (0.703-0.790) | 0.675 | 0.899 | 0.472 | | 0.665 (0.596-0.734) | 0.637 | 0.825 | 0.470 | | 0.692 (0.569-0.816) | 0.643 | 0.635 | 0.75 | |
| RS-BTI4-LC/NLC | 0.855 (0.821-0.889) | 0.779 | 0.786 | 0.803 | | 0.726 (0.662-0.789) | 0.667 | 0.600 | 0.752 | | 0.739 (0.629-0.850) | 0.691 | 0.5 | 0.862 | |
| RS-BTI6-LC/NLC | 0.807 (0.768-0.846) | 0.724 | 0.815 | 0.687 | | 0.708 (0.642-0.776) | 0.705 | 0.842 | 0.564 | | 0.739 (0.627-0.852) | 0.667 | 0.846 | 0.594 | |
| RS-BTI8-LC/NLC | 0.784 (0.744-0.825) | 0.694 | 0.584 | 0.841 | | 0.714 (0.648-0.780 ) | 0.709 | 0.842 | 0.581 | | 0.710 (0.587-0.838) | 0.738 | 0.885 | 0.531 | |
| RS-BM-LC/NLC | 0.762 (0.719-0.805) | 0.679 | 0.676 | 0.742 | | 0.699 ( 0.630-0.767) | 0.671 | 0.742 | 0.632 | | 0.659 (0.534-0.787) | 0.649 | 0.846 | 0.531 | |
| RS-BTI4-LC/NLC VS RS-BTI2-LC/NLC | | | | | 0.514 | | | | | 0.092 | | | | | 0.162 |
| RS-BTI4-LC/NLC VS RS-BTI6-LC/NLC | | | | | 0.067 | | | | | 0.726 | | | | | 0.984 |
| RS-BTI4-LC/NLC VS RS-BTI8-LC/NLC | | | | | 0.005* | | | | | 0.798 | | | | | 0.947 |
| RS-BTI4-LC/NLC VS RS-BM-LC/NLC | | | | | 0.001* | | | | | 0.562 | | | | | 0.654 |
| RS-BTI2-SC/NSC | 0.701 (0.739-0.850) | 0.710 | 0.843 | 0.634 | | 0.705 (0.612-0.797) | 0.650 | 0.793 | 0.532 | | 0.643 (0.489-0.798) | 0.616 | 0.88 | 0.556 | |
| RS-BTI4-SC/NSC | 0.910 (0.874-0.947) | 0.832 | 0.878 | 0.821 | | 0.856 (0.791-0.921) | 0.767 | 0.845 | 0.742 | | 0.750 (0.618-0.881) | 0.635 | 0.72 | 0.704 | |
| RS-BTI6-SC/NSC | 0.89 (0.846-0.926 ) | 0.794 | 0.861 | 0.740 | | 0.88 (0.755-0.899) | 0.717 | 0.707 | 0.806 | | 0.664 (0.513-0.814) | 0.654 | 0.56 | 0.778 | |
| RS-BTI8-SC/NSC | 0.867 (0.823-0.911 ) | 0.769 | 0.896 | 0.699 | | 0.789 (0.710-0.868) | 0.658 | 0.897 | 0.548 | | 0.711 (0.564-0.858) | 0.519 | 0.68 | 0.778 | |
| RS-BM-SC/NSC | 0.805 ( 0.749-0.861) | 0.697 | 0.765 | 0.764 | | 0.701 (0.607-0.796) | 0.642 | 0.534 | 0.806 | | 0.670 (0.508-0.831) | 0.614 | 0.52 | 0.895 | |
| RS-BTI4-SC/NSC VS RS-BTI2-SC/NSC | | | | | <0.001* | | | | | 0.003* | | | | | 0.04* |
| RS-BTI4-SC/NSC VS RS-BTI6-SC/NSC | | | | | 0.371 | | | | | 0.542 | | | | | 0.742 |
| RS-BTI4-SC/NSC VS RS-BTI8-SC/NSC | | | | | 0.130 | | | | | 0.196 | | | | | 0.214 |

| | | | | | | | | | | | | | | | |
|---|---|---|---|---|---|---|---|---|---|---|---|---|---|---|---|
| RS-BTI4-SC/NSC VS RS-BM-SC/NSC | | | | | 0.002* | | | | | 0.01* | | | | | 0.02* |
| RS-BTI2-BC/GIC | 0.779 (0.719-0.839) | 0.665 | 0.676 | 0.861 | | 0.709 (0.616-0.801) | 0.650 | 0.857 | 0.475 | | 0.694 (0.494-0.894) | 0.594 | 0.667 | 0.824 | |
| RS-BTI4-BC/GIC | 0.890 (0.848-0.932) | 0.811 | 0.829 | 0.820 | | 0.809 (0.730-0.888) | 0.744 | 0.643 | 0.869 | | 0.792 (0.618-0.966) | 0.686 | 0.867 | 0.768 | |
| RS-BTI6-BC/GIC | 0.864 (0.818-0.910) | 0.755 | 0.730 | 0.844 | | 0.745 (0.656-0.835 ) | 0.658 | 0.643 | 0.738 | | 0.780 (0.613-0.948) | 0.656 | 0.667 | 0.823 | |
| RS-BTI8-BC/GIC | 0.838 (0.789-0.888) | 0.742 | 0.856 | 0.648 | | 0.740 (0.650-0.830 ) | 0.667 | 0.839 | 0.525 | | 0.702 (0.501-0.903) | 0.719 | 0.600 | 0.882 | |
| RS-BM-BC/GIC | 0.786 (0.728-0.844) | 0.670 | 0.775 | 0.705 | | 0.737 ( 0.646-0.829) | 0.658 | 0.714 | 0.738 | | 0.667 (0.474-0.860) | 0.656 | 0.667 | 0.647 | |
| RS-BTI4-BC/GIC VS RS-BTI2-BC/GIC | | | | | 0.003* | | | | | 0.129 | | | | | 0.210 |
| RS-BTI4-BC/GIC VS RS-BTI6-BC/GIC | | | | | 0.371 | | | | | 0.299 | | | | | 0.453 |
| RS-BTI4-BC/GIC VS RS-BTI8-BC/GIC | | | | | 0.104 | | | | | 0.258 | | | | | 0.268 |
| RS-BTI4-BC/GIC VS RS-BM-BC/GIC | | | | | 0.001* | | | | | 0.178 | | | | | 0.186 |

AUC: area under the receiver operating characteristic curve; CI: confidence interval; ACC: accuracy; SEN: sensitivity; SPE: specificity; RS-BM, radiomics signature-brain metastasis area; RS-BTI, radiomics signature-brain-to-tumor interface area; RS-Com, radiomics signature-combined; *, $P<0.05$

**Table S5.** Morphological features of brain metastases

| Tumor type | T1CE intensity | T2W Intense | Perifocal edema | Solitary metastases | Homogeneity of CE accumulation | T1CE intensity | Central necrosis | Hemorr hage | Cystic appearance |
|---|---|---|---|---|---|---|---|---|---|
| **SCLC** | + | + | + | - | | + | | + | + |
| **NSCLC** | + | + | ++ | + | + | + | + | + | + |
| **BC** | + | + | + | - | | + | | + | + |
| **GIC** | + | ++ | ++ | - | + | + | + | + | - |

+ more likely/higher. -less likely/lower.

**References for Table**


1. **Zakaria R, Das K, Bhojak M, et al. The role of magnetic resonance imaging in the management of brain metastases: diagnosis to prognosis. Cancer Imaging 2014; 14:1-8.**
2. **Pekmezci M, Perry A. Neuropathology of brain metastases. Surg Neurol Int 2013; 4(Suppl 4):S245.**
3. **Hodler J, Von Schulthess GK, Zollikofer CL. Diseases of the brain, head & neck, spine: diagnostic imaging and interventional techniques. Springer 2007.**
4. **Dolgushin M, Kornienko V, Pronin I. Brain metastases: Advanced neuroimaging. Springer 2017.**
5. **Wang Q, Li Q, Mi R, et al. Radiomics nomogram building from multiparametric MRI to predict grade in patients with glioma: a cohort study. J Magn Reson Imaging 2019; 49:825-833.**
6. **Ahn SJ, Joo B, Park M, et al. Dural metastasis in breast cancer: MRI-based morphological subtypes and their clinical implications. Cancer Res Treat 2024; 56:1105-1112.**

Table S6. Comparison of model performance with and without ComBat harmonization

| | | Training cohort | | | | Internal validation cohort | | | | External cohort | | | |
|---|---|---|---|---|---|---|---|---|---|---|---|---|---|
| | RS | AUC | ACC | SEN | SPE | AUC | ACC | SEN | SPE | AUC | ACC | SEN | SPE |
| Non-combat | RS-BTI4-LC/NLC | 0.855 | 0.779 | 0.786 | 0.803 | 0.726 | 0.667 | 0.600 | 0.752 | 0.739 | 0.691 | 0.500 | 0.862 |
| | RS-BTI4-SC/NSC | 0.910 | 0.832 | 0.878 | 0.821 | 0.856 | 0.767 | 0.845 | 0.742 | 0.750 | 0.635 | 0.72 | 0.704 |
| | RS-BTI4-BC/GIC | 0.890 | 0.811 | 0.829 | 0.820 | 0.809 | 0.744 | 0.643 | 0.869 | 0.792 | 0.686 | 0.867 | 0.768 |
| Combat | RS-BTI4-LC/NLC | 0.831 | 0.781 | 0.777 | 0.775 | 0.713 | 0.717 | 0.652 | 0.787 | 0.736 | 0.712 | 0.616 | 0.827 |
| | RS-BTI4-SC/NSC | 0.892 | 0.802 | 0.892 | 0.804 | 0.832 | 0.732 | 0.829 | 0.715 | 0.722 | 0.650 | 0.700 | 0.694 |
| | RS-BTI4-BC/GIC | 0.883 | 0.798 | 0.800 | 0.782 | 0.795 | 0.700 | 0.684 | 0.833 | 0.781 | 0.670 | 0.849 | 0.751 |

**Table S7: Comparison of original lesion-level and patient-level performance of BTI-4 radiomics models**

| Classification task | Cohort | Patients, n | Class distribution, n/n | Original lesion-level AUC | Patient-level AUC (95% CI) | Delta AUC |
|---|---|---|---|---|---|---|
| LC vs. NLC | Training | 286 | 161/125 | 0.855 | 0.824 (0.772-0.873) | −0.031 |
| LC vs. NLC | Internal validation | 143 | 81/62 | 0.726 | 0.750 (0.662-0.832) | +0.024 |
| LC vs. NLC | External validation | 33 | 18/15 | 0.739 | 0.744 (0.611-0.952) | +0.005 |
| SCLC vs. NSCLC | Training | 161 | 54/107 | 0.910 | 0.897 (0.844-0.941) | −0.013 |
| SCLC vs. NSCLC | Internal validation | 81 | 28/53 | 0.856 | 0.815 (0.717-0.903) | −0.041 |
| SCLC vs. NSCLC | External validation | 18 | 8/10 | 0.750 | 0.733 (0.524-0.927) | −0.017 |
| BC vs. GIC | Training | 125 | 79/46 | 0.890 | 0.916 (0.858-0.963) | +0.026 |
| BC vs. GIC | Internal validation | 62 | 39/23 | 0.809 | 0.746 (0.606-0.861) | −0.063 |
| BC vs. GIC | External validation | 15 | 7/8 | 0.792 | 0.736 (0.572-0.895) | −0.056 |

## Supplementary Appendix S1

**MRI acquisition parameters across participating centers**

| Center | Scanner (3.0 T) | Sequence | FOV (mm) | Matrix size | Slice thickness (mm) | TR (ms) | TE (ms) |
|---|---|---|---|---|---|---|---|
| **Center 1** | **Philips Ingenia** | **T1CE** | **194 × 230** | **320 × 216** | **5.0** | **270** | **2.48** |
| | **(Netherlands)** | **T2W** | **194 × 230** | **384 × 227** | **5.0** | **3630** | **87** |
| **Center 2** | **Siemens Verio** | **T1CE** | **179 × 230** | **320 × 187** | **6.0** | **1400** | **9** |
| | **(Erlangen, Germany)** | **T2W** | **194 × 230** | **320 × 270** | **6.0** | **3500** | **99** |
| **Center 3** | **GE Discovery** | **T1CE** | **240 × 240** | **512 × 512** | **5.0** | **1750** | **24.0** |
| | **(USA)** | **T2W** | **240 × 240** | **512 × 512** | **5.0** | **4969** | **92.7** |

Radiomics Feature Extraction Workflow:
Prior to feature extraction, the original magnetic resonance (MRI) images undergo a series of normalization and enhancement procedures to improve the reliability and consistency of the subsequent radiomic analysis. The specific steps are as follows:
(1)Image Intensity Normalization:To standardize the intensity distribution across all MR images, voxel intensities are normalized based on the global mean and standard deviation of each image. This process ensures that the resulting intensity values follow a unified scale. A normalization scaling factor of 100 is applied to maintain the normalized intensities within a controlled dynamic range.Image Intensity.
(2)Voxel Resampling and Interpolation:To achieve spatial resolution consistency across different scans, all images are resampled to an isotropic voxel size of [1, 1, 1] $mm^3$. Cubic B-spline interpolation is employed during resampling to preserve structural integrity and minimize interpolation artifacts.
(3)Multifilter Transformations for High-Dimensional Feature Augmentation:
Before extracting texture and morphological features, MR images are processed through a series of image filters designed to enhance the diversity of feature representation. The following filters are applied:
1.Wavelet Transform: Decomposes the image into high- and low-frequency components along three spatial dimensions, producing eight wavelet sub-bands (e.g., HHH, LLL) to capture multiscale spatial textures.
2.Laplacian of Gaussian (LoG) Filtering: Enhances edge and texture details using Gaussian kernels with varying sigma values (1.0, 3.0, and 5.0), highlighting structures at different spatial scales.
3.Square and Square Root Transformations: Apply mathematical operations to the voxel intensities to amplify or compress specific ranges of signal distribution.
4.Logarithmic and Exponential Transforms: Enhance contrast and dynamic range by applying logarithmic or exponential functions to the absolute voxel intensity values.
5.Gradient Filtering: Computes the magnitude of local intensity gradients, thereby emphasizing spatial intensity changes and edge information.
6.Local Binary Pattern (LBP): Implements local texture encoding for both 2D and 3D contexts (e.g., lbp-2D, lbp-3D-k), enabling the characterization of fine-grained regional patterns within the image.

## Supplementary Appendix S2

The formulas of the developed radiomics signatures as follow:

RS-BTI4-LC/NLC=16.5155
-24.9960*lbp-2D_glszm_SizeZoneNonUniformity(T2W)
-16.6648*logarithm_gldm_DependenceNonUniformityNormalized(T1CE)
-16.2826*log-sigma-3-0-mm-3D_ngtdm_Contrast(T2W)+10.2147*wavelet-LLH_glszm_ZonePercentage(T1CE)
+6.7848*wavelet-LHL_glcm_MaximumProbability(T1CE)
-5.4342*wavelet-LHH_glszm_SizeZoneNonUniformityNormalized(T2W)
+4.1151*wavelet-LHH_glcm_MCC(T2W)
+3.9315*wavelet-LHH_glszm_GrayLevelNonUniformityNormalized(T2W)
-3.3051*wavelet-HLL_glcm_SumSquares(T2W)
+3.2100*square_glrlm_GrayLevelNonUniformityNormalized(T2W)
+2.8511*wavelet-HHL_glszm_SmallAreaLowGrayLevelEmphasis(T2W)
-2.3290*lbp-3D-k_glszm_SmallAreaEmphasis(T2W)
-2.1875*original_shape_Elongation(T2W)

RS-BTI4-SC/NSC = -26.7279
+79.0707*gradient_gldm_SmallDependenceLowGrayLevelEmphasis(T1CE)
+4.9464*lbp-2D_firstorder_Mean(T1CE)
-139.6013*wavelet-LHL_ngtdm_Coarseness(T1CE)
+5.2666*wavelet-LHL_glszm_GrayLevelNonUniformityNormalized(T1CE)
+0.6231*wavelet-LHL_firstorder_Skewness(T1CE)
+0.00003382*wavelet-LHL_glszm_LargeAreaEmphasis(T1CE)
-1.4977*exponential_ngtdm_Strength(T2W)
+15.8343*wavelet-LHH_glcm_Imc1(T2W)
+3.5269*log-sigma-1-0-mm-3D_glcm_MaximumProbability(T1CE)
+0.5860*lbp-2D_firstorder_10Percentile(T1CE)

RS-BTI4-GC/GIC = 4.5326
−2.3783*lbp-2D_glszm_ZoneEntropy(T2W)
+2.5626*wavelet-HLH_firstorder_Median(T1CE)
−15.3001*wavelet-LLL_glszm_GrayLevelNonUniformityNormalized(T2W)
−6.5349*wavelet-HHL_glszm_SmallAreaLowGrayLevelEmphasis(T2W)
+0.2123*wavelet-LLH_ngtdm_Strength(T1CE)
+6.3386*square_glszm_SmallAreaLowGrayLevelEmphasis(T1CE)
+2.9284*log-sigma-3-0-mm-3D_glszm_SmallAreaEmphasis(T1CE)
−76.9018*lbp-3D-k_glszm_ZonePercentage(T2W)
+0. 1901*wavelet-LLL_gldm_DependenceVariance(T2W)

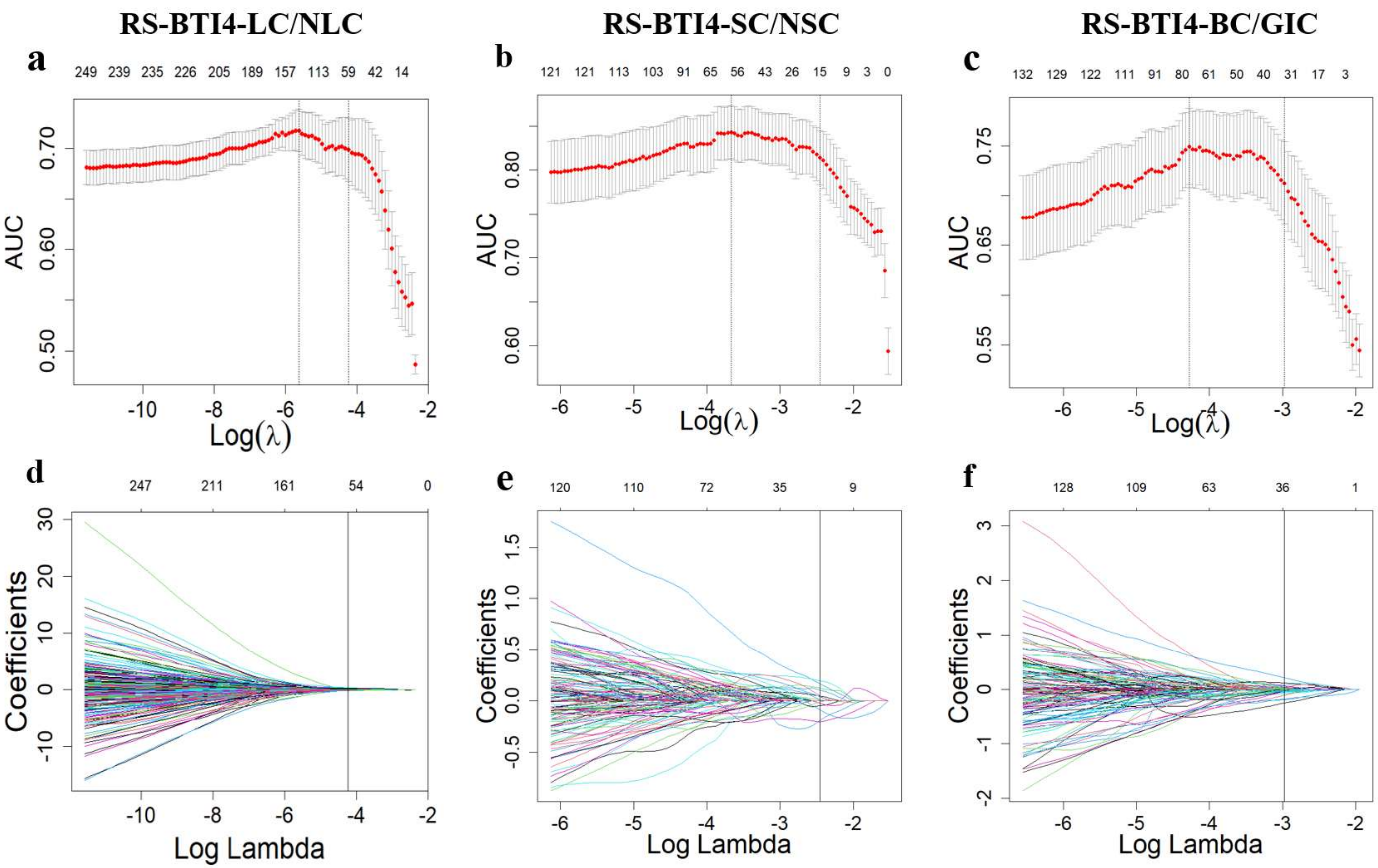


**Figure S1.**Radiomic features selection based on 4 mm BTI were analyzed using LASSO logistic regression. LASSO regression was used to analyze feature coefficients with 10-fold cross-validation. RS-BTI 4-LC/NLC (a), RS-BTI 4-SC/NSC (b), and RS-BTI 4-BC/GIC (c) are the best logarithms screened (Lambda). coefficients plotted for RS-BTI 4-LC/NLC (d), RS-BTI 4-SC/NSC (e), and RS-BTI 4-BC/GIC (f) as logarithmic (Lambda) sequences.

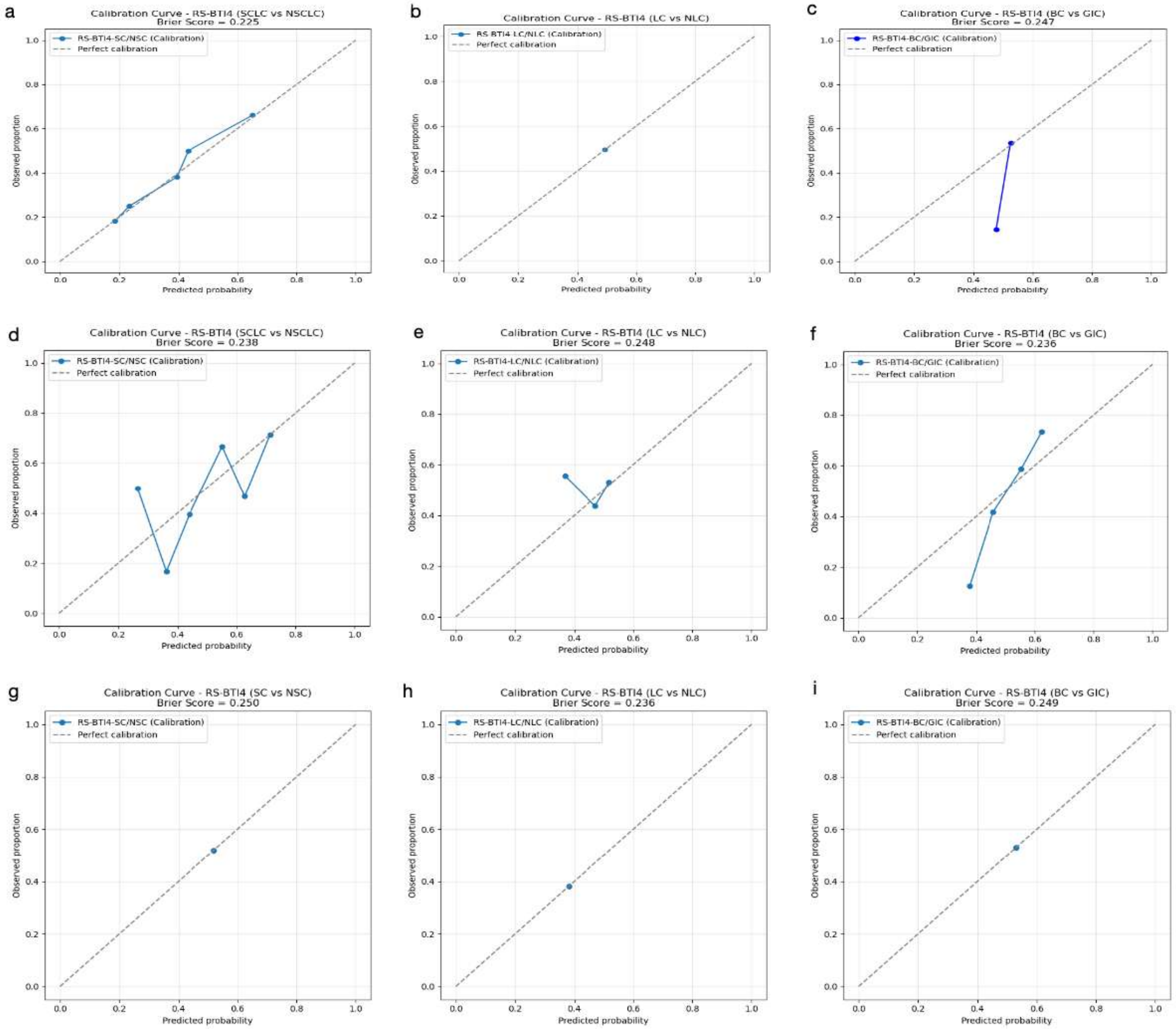


**Figure S2.** Calibration curves of the RS-BTI4 models for three classification tasks: RS-BTI4-SC/NSC, RS-BTI4-LC/NLC, and RS-BTI4-BC/GIC.(a–c) Training cohort; (d–f) internal validation cohort; (g–i) external validation cohort. Each curve compares the predicted probability against the observed proportion. The dashed diagonal line represents perfect calibration, while the solid line represents the model's performance. The Brier scores are displayed within each panel.

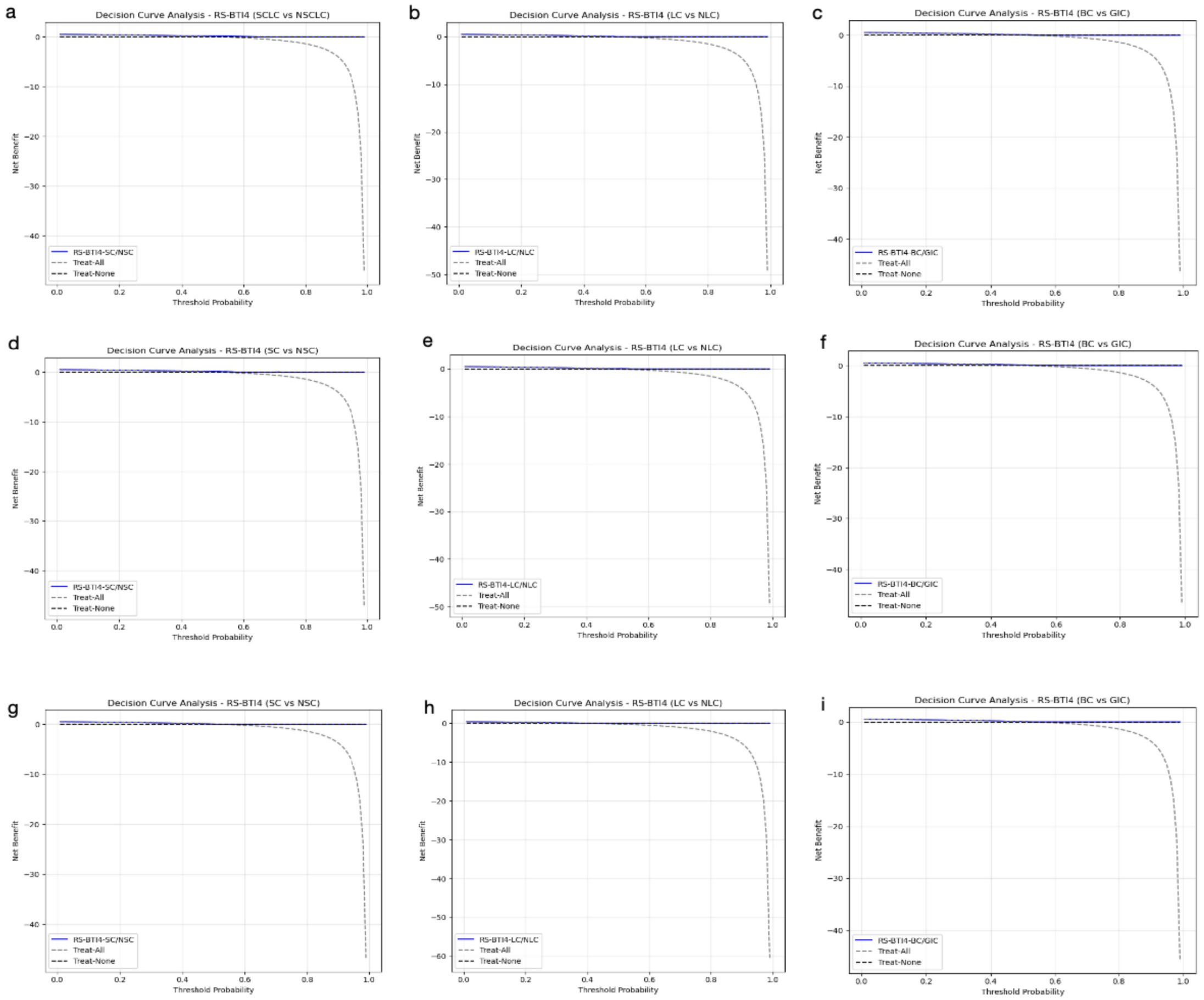


**Figures S3.** Decision curve analyses of the RS-BTI4 models for three classification tasks: RS-BTI4-SC/NSC, RS-BTI4-LC/NLC, and RS-BTI4-BC/GIC.(a–c) Training cohort; (d–f) internal validation cohort; (g–i) external validation cohort.Each curve demonstrates the clinical net benefit of the RS-BTI4 models across a range of threshold probabilities, compared with the “treat-all” and “treat-none” strategies. The higher net benefit of the RS-BTI4 models indicates their potential clinical utility in identifying brain metastasis origins.
